# Proximale Femurresektion mit endoprothetischer Rekonstruktion bei malignen Knochentumoren: Operative Technik über den vorderen Hüftzugang

**DOI:** 10.1007/s00132-025-04720-w

**Published:** 2025-09-16

**Authors:** Dietmar Dammerer, Melanie Ardelt, Gianpaolo Leone, Martin Thaler, David Putzer, Hannes Stofferin, Johannes Neugebauer

**Affiliations:** 1 Abteilung für Orthopädie und Traumatologie, Universitätsklinikum Krems, Mitterweg 10, 3500 Krems an der Donau, Österreich; 2 Department of Orthopaedics & Traumatology, University Hospital Krems, Karl Landsteiner University of Health Sciences, Mitterweg 10, 3500 Krems, Österreich; 3 Center for Regenerative Medicine and Orthopaedics, University for Continuing Education Krems, Dr. Karl-Dorrek-Str. 30, 3500 Krems, Österreich; 4 Klinik und Poliklinik für Orthopädie und orthopädischer Chirurgie der Universitätsmedizin Greifswald, Universität Greifswald, Ferdinand-Sauerbruch-Straße 17489, Greifswald, Deutschland; 5 Endoprothetikzentrum München West, Helios Klinikum, Steinerweg 5, 81241 Munich, Deutschland; 6 Experimentelle Orthopädie, Universitätsklinik für Orthopädie und Traumatologie, Medizinische Universität Innsbruck, Anichstraße 35, 6020 Innsbruck, Österreich; 7 Institut für Klinisch-Funktionelle Anatomie, Medizinische Universität Innsbruck, Müllerstraße 59, 6020 Innsbruck, Österreich

**Keywords:** Femur, Hüftprothesenimplantation, Maligne Neoplasien, Osteotomie, Sarkom, Femur, Hip prosthesis implantation, Malignant neoplasm, Osteotomy, Sarcoma

## Abstract

**Operationsziel:**

Die proximale Femurresektion mit EPR bedeutet die chirurgischen Tumorentfernung mit dem Ziel der R0-Resektion bei malignen Knochentumoren, nach Möglichkeit (in Abhängigkeit vom Tumor) unter Schonung der umgebenden Weichteil- und neurovaskulären Strukturen sowie der funktionellen Wiederherstellung der Hüftgelenksstabilität.

**Indikationen:**

Maligner Knochentumor (Sarkom) des proximalen Femurs, pathologische Fraktur (Metastase/Knochenstoffwechselstörung), Rezidivtumor, (Karzinom‑)Metastase im proximalen Femur.

**Kontraindikationen:**

Ausgedehnte Weichteilinfiltration mit unzureichender Rekonstruktionsmöglichkeit (Weichteildeckung), generalisierte Metastasierung ohne kurative Behandlungsoption (Lebensdauer begrenzt), Infektion im Operationsgebiet, kritischer Allgemeinzustand des Patienten, der eine große Operation nicht erlaubt.

**Operationstechnik:**

Der Hautschnitt erfolgt longitudinal unter Einbezug der Biopsienarbe. An dieser Stelle soll erneut auf die Wichtigkeit korrekter Biopsien hingewiesen werden, da sie den Operationszugang vorgeben. Schon die Biopsie sollte im ausgewiesenen Zentrum durchgeführt werden. Nach schrittweiser Weichteilpräparation und Schonung der neurovaskulären Strukturen erfolgt die Osteotomie des Femurs. Die Hüftkapsel sollte nach Möglichkeit erhalten und rekonstruiert werden. Anschließend wird das Tumorresektat entnommen, die prothetische Versorgung mit einer modularen Tumorprothese durchgeführt und die Weichteile rekonstruiert, um eine optimale Stabilität zu gewährleisten.

**Weiterbehandlung:**

Postoperativ erfolgt eine frühfunktionelle Mobilisation mit Teilbelastung. Eine adjuvante Therapie wird individuell nach Tumorstadium geplant. Regelmäßige radiologische Kontrollen sichern den langfristigen Erfolg.

**Evidenz:**

Die EPR nach Tumorresektion ist ein etabliertes Verfahren mit guter funktioneller Wiederherstellung und onkologischer Sicherheit. Langzeitstudien zeigen zufriedenstellende funktionelle Ergebnisse und vertretbare Komplikationsraten.

**Video online:**

Die Online-Version dieses Beitrags enthält ein Video zur proximalen Femurresektion mit endoprothetischer Rekonstruktion bei malignen Knochentumoren. Dieses Supplementary-Material finden Sie unter 10.1007/s00132-025-04720-w oder unter https://www.springermedizin.de/die-orthopaedie

## Abstract (en)

**Objective of the surgery:**

Proximal femur resection with EPR aims to achieve oncological tumor removal while preserving surrounding soft tissue and neurovascular structures, ensuring functional restoration of hip joint stability.

**Indications:**

Malignant bone tumors of the proximal femur, pathological fractures due to tumor involvement, recurrent tumors after previous resection, extensive destruction of the proximal femur due to metastases.

**Contraindications:**

Extensive soft tissue infiltration with inadequate reconstruction potential, generalized metastases without curative treatment options, severe infections in the surgical area, critical general condition prohibiting major surgery.

**Surgical technique:**

A longitudinal skin incision is made, incorporating the biopsy scar. After sequential soft tissue preparation and preservation of neurovascular structures, femoral osteotomy is performed according to preoperative planning. The hip capsule is preserved and reconstructed. The tumor resection is followed by endoprosthetic reconstruction with a modular tumor prosthesis and subsequent soft tissue reconstruction to ensure optimal stability.

**Follow-up:**

Postoperative management includes early functional mobilization with partial weight-bearing. Adjuvant therapy is planned individually based on tumor staging. Regular radiological follow-up is essential for long-term success.

**Evidence:**

EPR following tumor resection is an established procedure with good functional outcomes and oncological safety. Long-term studies demonstrate satisfactory functional results and acceptable complication rates.

## Allgemeines

Die Inzidenz von Krebserkrankungen steigt aufgrund der alternden Bevölkerung. In Österreich ist die Prävalenz von Krebserkrankungen zwischen dem Jahr 2014 und 2024 um 24 % gestiegen [1]. Die weltweit geschätzte Inzidenz primär maligner Knochentumoren liegt bei 5 Fällen pro 100.000 Einwohnern pro Jahr. Global gesehen ist das nicht mehr als 1 % aller Krebserkrankungen [2]. Chirurgisch relevant ist die Tatsache, dass neben den primär malignen Knochentumoren (Sarkomen), welche nur circa ein Fünftel bis ein Sechstel aller Sarkome ausmachen, auch Karzinome am dritthäufigsten in das Skelettsystem absiedeln. Knochentumoren sind zu etwa zwei Drittel Metastasen und nur zu einem Drittel ossären Ursprungs – etwa jeder 3.–5. Krebspatient entwickelt derartige Absiedlungen [3]. Unter den knöchernen Sarkomen hat das Osteosarkom mit 0,2 Fällen/100.000 Einwohnern/Jahr die höchste Inzidenz [2].

Tumoren und Metastasen haben bei Auftreten in den unteren Extremitäten eine höhere klinische Bedeutung, da hier von pathologischen Frakturen gewichtstragende Knochen betroffen sind. Schmerzen und Funktionsverlust können in allen Extremitäten resultieren. Je nach Ausmaß, Morphologie und Entität kann als Ultima Ratio die Amputation als einzig überlebenssichernd notwendig werden [4].

Therapeutisch stehen verschiedene chirurgische Verfahren zur Verfügung. Hierzu zählen Osteosyntheseverfahren: intramedulläre Nagelung, Plattenosteosynthesen, Verbundosteosynthesen mit Knochenzement sowie endoprothetische Verfahren wie Hemiprothesen, totale Endoprothesen oder modulare Tumor‑/Revisionsendoprothesen. Die Versorgung ist abhängig vom Ausmaß des Knochenverlustes und der Weichgewebsdeckung [5].

Dieses Manuskript präsentiert die Resektion des proximalen Femurs mit endoprothetischer Rekonstruktion (EPR) durch den vorderen Hüftzugang. Dieser anspruchsvolle, jedoch an Popularität zunehmende Eingriff birgt neben onkologischen auch technische Herausforderungen und bringt meist ein annehmbares funktionelles Ergebnis [5]. Die Wiederherstellung der Gelenkfunktion in Kombination mit Schmerzarmut und Freiheit bieten eine Verbesserung der Lebensqualität. In der Regel ist eine postoperative Vollbelastung erlaubt, sodass alltägliche Aktivitäten wieder aufgenommen werden können.

Trotz aller chirurgischen Mühen sowie industriellen und pharmakologischen Fortschrittes ist dieser Eingriff nach wie vor komplikationsbehaftet und sollte daher nur in ausgewiesenen Zentren durchgeführt werden. Potenzielle Komplikationen sind verlängerte Wundsekretion und Serombildungen aufgrund von Weichteilverlusten. Dem Weichteilmanagement kommt in der Revisions- und Tumorendoprothetik eine große Bedeutung zu. Periprothetische Gelenksinfektionen (PJI) bei Tumor‑/Megaprothesen werden in der Literatur mit bis zu 15 % angegeben [6, 7]. Diese können schwerwiegende Folgen wie Multiorganversagen oder ein septisches Schocksyndrom nach sich ziehen [8–11].

Dahinter kommen aseptische Lockerungen, Becken- und Beinvenenthrombosen, erhöhte Luxationstendenz sowie periprothetische Frakturen [8, 12, 13]. All diese genannten Faktoren haben neben der medizinischen Bedeutung auch einen wirtschaftlichen Aspekt [14].

Ziel dieser Publikation ist es, die Indikation, Kontraindikationen sowie die Operationstechnik und Nachbehandlung anhand eines Patientenfalles nach proximaler Femurresektion und EPR wegen eines malignen Knochentumors darzustellen.

## Definition und Operationsindikation

Die proximale Femurresektion mit EPR ermöglicht die chirurgische Entfernung von Knochentumoren oder metastatisch veränderten Knochenanteilen mit funktioneller Wiederherstellung des Hüftgelenks. Ziel ist die Schaffung einer stabilen, belastbaren Gelenksituation bei maximaler Schonung von Muskeln und neurovaskulären Strukturen.

Die Indikation zum proximalen Femurersatz bei Knochenmetastasen richtet sich nach der Tumorausbreitung und dem klinischen Zustand des Patienten. Eine radikale Resektion mit EPR wird bevorzugt bei singulären oder oligometastatischen Befunden durchgeführt, wenn dadurch eine dauerhafte Schmerzlinderung, Stabilität und Mobilität erzielt werden kann. Bei multiplen Metastasen wird die Operation meist im Rahmen einer palliativen Versorgung mit dem Ziel der Schmerzreduktion und Verbesserung der Lebensqualität eingesetzt, sofern der Allgemeinzustand des Patienten eine Operation erlaubt und eine Lebenserwartung von mehr als 3 Monaten besteht.

### Vorteile

- *Sofortige Belastbarkeit*: Die Endoprothese erlaubt eine frühzeitige Vollbelastung.
- *Erhalt der Mobilität*: Durch die prothetische Rekonstruktion kann eine frühe Mobilisation erfolgen.
- *Stabile Gelenkfunktion*: Die Rekonstruktion der Hüftkapsel, von muskulären Strukturen sowie die Implantatwahl minimieren das Luxationsrisiko.

### Nachteile

- *Erhöhtes Infektionsrisiko*: Endoprothesen sind anfälliger für periprothetische Infektionen.
- *Prothesenlockerung*: Erhöhtes Risiko für septische/aseptische Lockerung oder mechanische Komplikationen.
- *Eingeschränkte biologische Integration*: Im Gegensatz zu biologischen Rekonstruktionen (z. B. Allograft oder autologem vaskularisiertem Fibulatransplantat) keine knöcherne Heilung.

### Kontraindikationen

- Stark eingeschränkter Allgemeinzustand, der eine große Operation nicht erlaubt.
- Weichteildefizite, die eine stabile Weichteildeckung der Prothese nicht gewährleisten.
- Infektionen im Operationsgebiet, die das Risiko für Protheseninfektionen erhöhen.

## Fallbeschreibung

**Abb. 1:**
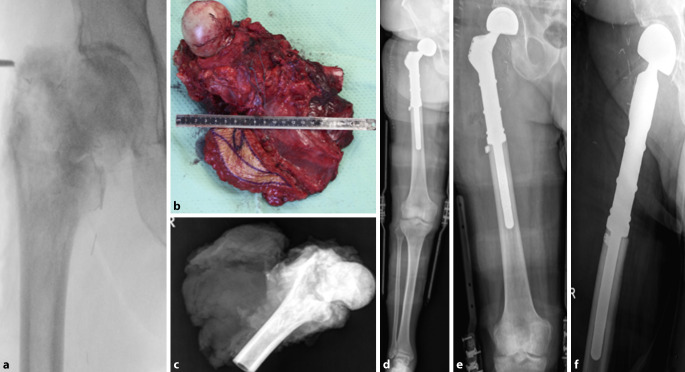
**a** Röntgen präoperativ a. p., **b** intraoperatives Bild des Tumors, **c** Röntgen des Resektats, **d** Ganzbeinstandaufnahme postoperativ a. p., **e** Röntgen postoperativ a. p., **f** Röntgen postoperativ axial

Ein 77-jähriger männlicher Patient wurde aufgrund eines multiplen Myeloms mit fortschreitender Osteolyse im proximalen Femur rechts zur weiteren Behandlung vorgestellt (Abb. 1a). In der Anamnese fand sich eine Vorbehandlung mittels Radio- und Chemotherapie. Aufgrund der Größenzunahme der Läsion und resultierender Frakturgefahr wurde eine Operation mittels modularer Tumorendoprothese (MUTARS® der Firma Implantcast, Buxtehude, Deutschland) durchgeführt. Die Beurteilung der Frakturgefahr erfolgte anhand des Mirel’s-Scores. Anhand dessen wurde eine hohe Wahrscheinlichkeit berechnet und die Indikation zur Stabilisierung gestellt. Er weist eine Sensitivität von 88 % für drohende pathologische Frakturen auf [15].

Die Operation wurde in Allgemeinanästhesie mit arterieller und zentralvenöser Katheteranlage durchgeführt. Die Implantatwahl erfolgte unter Berücksichtigung der anatomischen Gegebenheiten und einer notwendigen langfristigen Stabilität (Abb. 1b). Ergänzend kam das Super-Cable® Iso-ElasticTM-Cerclage-System (Kinamed, Camarillo, CA, USA) zum Einsatz, das durch seine elastischen Eigenschaften eine sichere Fixation der Knochenfragmente ermöglichte, ohne die Durchblutung des Knochens zu beeinträchtigen. Die Hüftrekonstruktion wurde mit einem Titan-Hüftkopf (Konus 12/24, Durchmesser 28 mm) durchgeführt, der in Kombination mit einem Restoration®-Insert (IDMM 28 mm) eine optimale Artikulation sicherte. Zur zusätzlichen Stabilisierung der rekonstruierten Hüftpfanne wurde eine Tritanium®-hemisphärische Cluster-Hole-Schale mit einem Durchmesser von 54 mm implantiert. Der Femurschaft wurde als zementfreie Variante mit einem Durchmesser von 15 mm und einer Länge von 120 mm gewählt, um eine stabile Verankerung im verbliebenen Knochen zu gewährleisten. Für die proximale Femurrevision kam eine silberbeschichtete 50/127°-Komponente zum Einsatz, die mit einer zusätzlichen „safety screw“ fixiert wurde, um die biomechanische Belastbarkeit zu erhöhen (Abb. 1d–f).

Am selbigen Tag konnte der Patient extubiert werden. Der weitere postoperative Verlauf gestaltete sich komplikationslos. Der Patient konnte am 9. postoperativen Tag entlassen werden.

Ein weiterer 69-jähriger männlicher Patient wurde aufgrund eines Osteosarkoms im Bereich des proximalen Femurs rechts zur weiteren Behandlung vorgestellt. Aufgrund der Erkrankung erfolgte eine Femurresektion mit anschließender endoprothetischer Versorgung.

Die Operation wurde in Allgemeinanästhesie mit arterieller und zentralvenöser Katheteranlage durchgeführt. Die Implantatwahl erfolgte unter Berücksichtigung der anatomischen Gegebenheiten und der notwendigen langfristigen Stabilität. Zur zusätzlichen Stabilisierung der rekonstruierten Hüftpfanne wurde eine Multipolar® Bipolar Cup (Fa. Zimmer, Warsaw, IN, USA) implantiert. Der Femurschaft wurde als zementfreie Variante gewählt, um eine stabile Verankerung im verbliebenen Knochen zu gewährleisten.

Am selben Tag konnte der Patient extubiert werden. Der weitere postoperative Verlauf gestaltete sich komplikationslos. Der Patient wurde am 14. postoperativen Tag in stabilem Zustand aus der stationären Behandlung entlassen.

## Operationstechnik

Der Patient liegt in Rückenlagerung auf dem Operationstisch. Die Beine werden neutral gelagert, um die Leiste freizuhalten. Nach der sterilen Desinfektion, die auch die Leistenregion umfasst, erfolgt die Abdeckung. Dabei wird darauf geachtet, dass die Leiste nicht abgedeckt wird, um bei Bedarf einen schnellen Gefäßzugang oder eine Blutstillung zu ermöglichen.

Der Hautschnitt wird longitudinal angelegt, beginnend etwas distal der Spina iliaca anterior superior bis zum Trochanter major, wobei die vorhandene Biopsienarbe in Schiffchenform ausgeschnitten wird (00:51). Dies ist vor allem bei Knochensarkomen relevant, weniger bei Karzinommetastasen. Falls eine totale Femurresektion erforderlich ist, kann der Schnitt lateral der Patella bis zum Kniegelenk distal bis zur Tuberositas tibiae erweitert werden. Das Risiko einer Läsion des N. cutaneus femoris lateralis kann verringert werden, wenn der Hautschnitt nach proximal leicht nach dorsal verlaufend angelegt wird – analog zum klassischen lateralen Hüftzugang.

Nach Darstellung der Fascia latae wird der N. cutaneus femoris lateralis sichtbar (01:37). Die weitere Präparation erfolgt durch Ablösung der Mm gluteii, wobei die Sehne des M. gluteus medius freigelegt wird (01:47). In diesem dargestellten Fall reicht die Tumorinfiltration bis an den Trochanter major, sodass eine Trochanter-major-Osteotomie nicht durchgeführt werden kann. Die Gluteus-medius-Sehne wird angeschlungen, um sie für die spätere Rekonstruktion zu nutzen (02:40).

Es wird nun eine schematische Darstellung der Trochanter-major-Osteotomie im anatomischen Präparat dargestellt. Durch dieses Manöver können sowohl die gluteale Muskulatur als auch die Außenrotatoren geschont und später für die Rekonstruktion verwendet werden (02:56). Im onkologischen Setting ist jedoch zu beachten, dass durch die Eröffnung des ossären Kompartiments das Risiko einer Tumorkontamination besteht. Da viele Tumoren im meta- bis epiphysären Bereich lokalisiert sind, ist der Sicherheitsabstand in diesen Fällen oftmals kritisch. Die Trochanterosteotomie muss daher sorgfältig geplant und individuell abgewogen werden. Im gezeigten Fall erfolgt die Darstellung ausschließlich am anatomischen Präparat – eine Osteotomie wurde im Rahmen der Operation nicht durchgeführt.

In weiterer Folge werden die A. perforantes dargestellt, geklippt und durchtrennt (03:45). Das Lumen der Gefäße sowie die verbliebene Biopsiestelle sind deutlich erkennbar (04:08).

Die weitere Präparation erfolgt dorsal mit knochennahem Ablösen der Muskulatur (04:15). Nach Spaltung der Faszie wird das neurovaskuläre Bündel freigelegt, einschließlich der A. femoralis und des Abgangs der A. profunda femoris (04:30). Diese werden unterfahren und zur Ligatur vorbereitet, wobei Epipontfäden verwendet werden (04:39). Die A. profunda wird mit dem Schmieden unterfahren und durchtrennt (05:12).

Strukturdarstellung am anatomischen Präparat (05:32): Es werden folgende Strukturen sichtbar: M. sartorius, N. femoralis, A. femoralis, A. profunda femoris, A. circumflexa femoris medialis, V. femoralis, M. pectineus, M. adductor sowie die A. circumflexa femoris lateralis mit ihren Ästen R. ascendens und R. transversus.

Vorbereitung der Femurosteotomie (06:40): Nach Weichteilpräparation durch den M. vastus lateralis wird das Femur mit dem Raspatorium freigelegt, um die Hohmann-Hebel zu platzieren. Wenn keine große Weichteilkomponente vorhanden ist, kann der Vastus lateralis zu großen Anteilen geschont werden, indem am lateralen Rand des Vastus lateralis die Präparation auf das Femur erfolgt. Nach präoperativer Planung der Resektionshöhe erfolgt die Resektion und Osteotomie des Femurs mit einer oszillierenden Säge (07:13). Anschließend werden Gewebeproben aus dem distalen Femur entnommen. Die Muskulatur wird nun dorsalseitig und im Bereich der Adduktoren abgelöst, bevor das Hüftgelenk eröffnet wird (07:30). Die Hüftkapsel wird längs gespalten und türflügelartig abgelöst, um eine optimale Weichteildeckung für die Prothese zu gewährleisten (07:55). Die Einhaltung der onkologischen Resektionsgrenzen hat dabei höchste Priorität (Abb. 1b, c). Nach Ablösung der Hüftgelenkskapsel dorsal und lateral wird das Resektat entnommen (08:31), wobei der N. ischiadicus zu sehen ist und die neurovaskulären Strukturen geschont wurden (08:56).

Prothetische Versorgung (09:05): Nach Kantenbearbeitung und Einzeichnung der Rotation erfolgt die Sicherung mit einer Cerclage gemäß Operationsanleitung (09:15). Das Femur wird für das Einbringen der Raspel vorbereitet – ein Schritt, für den besondere Sorgfalt empfohlen wird. Anschließend wird das Probeimplantat gemäß der Resektatlänge vorbereitet und eingebracht (09:35), gefolgt von der Einstellung der Anteversion und Fixierung mit Schrauben gemäß Operationsanleitung. Als intraoperative Kontrolle der gewählten Länge wird empfohlen, das Probeimplantat direkt neben das entnommene Resektat zu legen und die Übereinstimmung visuell zu überprüfen.

Nach erfolgreicher Probereposition wird die definitive Prothese eingebracht, einschließlich eines Trevira-Schlauchs mit Tabaksbeutelnaht distal (10:02). Die rekonstruierte Hüftgelenkskapsel mit den entsprechenden Muskelansätzen ist dargestellt (10:30). Nach Aufbringen des Duokopfes und Reposition zeigt sich eine luxationsfreie und stabile Hüftgelenkssituation.

Weichteilrekonstruktion (11:06): Nun folgt die Deckung der Prothese durch Rekonstruktion der Hüftgelenkskapsel und Anpassung des Trevira-Schlauchs. Die Abduktormuskulatur wird an den Trevira-Schlauch sowie an die Prothese angeschlungen, was eine stabile Weichteildeckung gewährleistet.

Zum Abschluss erfolgten eine Totraumverkleinerung und Rekonstruktion der Muskulatur mit einer fortlaufenden Naht, um biomechanische Stabilität zu gewährleisten (11:50). Eine Redon-Drainage mit Sog wird eingelegt, gefolgt vom Hautverschluss mit intrakutanen Fäden und Hautklammern (Abb. 1d–f).

## Postoperative Behandlung

### Erster Patient.

Als Infektprophylaxe erhielt der Patient Cefuroxim 1,5 g bis zum 7. postoperativen Tag.

Eine Kryotherapie wurde während des stationären Aufenthalts angewendet, um Schwellungen und postoperative Schmerzen zu reduzieren. Die Wunddrainage wurde am 4. postoperativen Tag entfernt, nachdem die Fördermenge unter 80 ml gesunken war.

Die Mobilisation begann frühzeitig unter physiotherapeutischer Anleitung bei Anwendung einer Teilbelastung von 50 % des Körpergewichts an zwei Unterarmgehstützen. Zusätzlich wurde eine Kniegelenksorthese (DONJOY® 4Titude®, Enovis Corporation, Wilmington, Deutschland) in 0/0/0-Stellung zur Stabilisierung eingesetzt. Nach 6 Wochen konnte eine Vollbelastung durchgeführt werden.

### Zweiter Patient.

Hier erhielt der Patient Cefuroxim 1,5 g bis zum 14. postoperativen Tag. Die Redon-Drainagen wurden am 5. postoperativen Tag entfernt.

Die Mobilisation begann frühzeitig unter physiotherapeutischer Anleitung unter Anwendung einer Vollbelastung an zwei Unterarmgehstützen. Zusätzlich wurde eine Europahose-Orthese angelegt sowie bei N.-femoralis-Schwäche rechts eine Kniegelenksorthese verwendet.

Beiden Patienten wurde nach der stationären Behandlung eine weiterführende Rehabilitation empfohlen. Diese umfasste physiotherapeutische Maßnahmen zur Verbesserung der Muskelkraft, Gelenkmobilität und Koordination sowie ein gezieltes Gehtraining.

Die durchschnittliche Heilungszeit bei solchen Eingriffen beträgt etwa 3–6 Monate, abhängig vom individuellen Heilungsverlauf und möglichen Komplikationen.

Die Arbeitsfähigkeit ist in diesen Fällen nicht relevant. Bei berufstätigen Patienten hängt sie von der körperlichen Belastung der Tätigkeit ab und ihre Wiedererlangung kann, insbesondere bei schwerer körperlicher Arbeit, mehrere Monate betragen.

## Fehler, Gefahren, Komplikationen

Eine erfolgreiche proximale Femurresektion mit EPR setzt eine sorgfältige präoperative Planung, eine präzise intraoperative Durchführung und ein konsequentes postoperatives Management voraus. Dennoch können zahlreiche Komplikationen auftreten, die es frühzeitig zu erkennen und gezielt zu behandeln gilt.

### Präoperative Risiken und Management

Die Grundvoraussetzung für eine radikale Metastasenresektion und EPR ist ein guter Allgemeinzustand des Patienten sowie eine Lebenserwartung von mehr als 3 Monaten [9].

Ein zentrales Element der präoperativen Planung ist die Einbindung des Patienten in ein interdisziplinäres Setting sowie die Vorstellung im Tumorboard. Durch die enge Zusammenarbeit verschiedener Fachdisziplinen wie Onkologie, Radiologie, Gefäßchirurgie und Anästhesie können Therapieoptionen individuell abgestimmt und potenzielle Risiken frühzeitig erkannt werden. Das Tumorboard unterstützt eine strukturierte, leitliniengerechte Entscheidungsfindung unter Berücksichtigung patientenspezifischer Faktoren.

Darauf aufbauend ist eine umfassende präoperative Bildgebung essenziell, um den Operationszugang zu planen und anatomische Strukturen bestmöglich zu visualisieren. Sie ermöglicht die Darstellung von Gefäß- und neurovaskulären Strukturen, wodurch eine potenzielle Notwendigkeit eines Gefäßersatzes frühzeitig erkannt werden kann.

### Intraoperative Risiken und Management

Die Wahl zwischen einem zementierten und einem zementfreien Femurschaft erfolgt patientenindividuell in Abhängigkeit von der Knochenqualität, dem Alter und der verbleibenden Femurlänge. Bei ausreichend stabiler Kortikalis und guter diaphysärer Verankerungsmöglichkeit bevorzugen wir einen zementfreien Schaft. Liegen hingegen eine reduzierte Knochenqualität – etwa bei älteren Patienten oder tumorbedingter Osteolyse – oder eine sehr kurze Restdiaphyse vor, kommt ein zementierter Schaft zum Einsatz, um eine zuverlässige Primärstabilität zu gewährleisten [16].

Die Pfannenwahl richtet sich nach dem Zustand des Azetabulums: Bei intakter Pfanne kann eine bipolare Pfanne eingesetzt werden, die zusätzlich vor Luxationen schützt. Liegt eine Metastasierung des Azetabulums vor, ist eine zementierte Pfanne indiziert.

Zur Reduktion des periprothetischen Frakturrisikos ist die Anlage von Cerclagen sinnvoll. Weitere intraoperative Risiken umfassen Gefäßverletzungen oder Gefäßverschlüsse, insbesondere durch anhaltenden Zug auf Gefäße oder deren Umstechung [17]. Die A. femoralis profunda sollte stets klar dargestellt werden, um eine Ligatur bei Blutungen rechtzeitig vornehmen zu können.

### Postoperative Risiken und Management

Zu den seltenen, aber schwerwiegenden postoperativen Komplikationen zählen Paresen des N. femoralis und N. ischiadicus, die sich als N.-peroneus-Paresen manifestieren können [18, 19]. Bei einem vollständigen sensomotorischen Funktionsverlust, insbesondere nach Anlage einer Drahtcerclage, ist eine offene Revision erforderlich.

Ein weiteres relevantes Problem ist die verzögerte Wundsekretion, die in bis zu 48 % der Fälle auftritt [20]. Eine okklusive Wundverschlussmethode, wie im Operationsvideo gezeigt, kann das Risiko einer PJI erheblich senken.

Die Luxationsrate nach proximaler Femurresektion ist mit 37 % außergewöhnlich hoch [21]. Ursachen hierfür können mehrere Faktoren sein: Einerseits eine gestörte Balance zwischen Abduktoren und M. iliopsoas, insbesondere bei kompartimentübergreifenden Tumorresektionen mit Weichteilresektion. Andererseits eine unzureichende Kapsel- und Abduktorenrekonstruktion sowie fehlende Refixation der pelvitrochantären Muskulatur, die für die Gelenkstabilität und die Wiedererlangung der Abduktionsfähigkeit essenziell ist. Der Einsatz eines Trevira-Schlauches kann die Luxationsrate senken.

Die Rezidivrate beträgt 7 % [21] und erfordert eine engmaschige Nachsorge.

### Fazit

Eine sorgfältige Planung, präzise intraoperative Durchführung und konsequente postoperative Überwachung sind essenziell, um das Komplikationsrisiko zu minimieren und die Funktionalität sowie Lebensqualität der Patienten langfristig zu erhalten.

## Evidenz der Technik

Die in dieser Arbeit beschriebene Technik wird in der vorhandenen Literatur nur selten dokumentiert. Hervorzuheben ist, dass in der vorliegenden Technik der anteriore Zugang für die proximale Femurresektion genutzt wurde. Üblicherweise erfolgen proximale Femurersatzverfahren in Seitenlage über einen lateralen Zugang. Die Anwendung des anterioren Zugangs stellt daher eine abweichende und besondere Herangehensweise dar, die potenzielle Vorteile hinsichtlich Weichteilschonung und postoperativer Mobilisation bieten kann. Ähnliche operative Strategien und Implantatentscheidungen finden sich in verschiedenen Studien, die wesentliche Erkenntnisse zur Optimierung der Behandlung liefern.

### Implantatauswahl und Luxationsprävention

Laut Guzik et al. basiert die Wahl des Implantats auf der Knochenqualität: In einer Kohorte von 75 Patienten wurden in 53 Fällen zementfreie Schäfte und in 68 Fällen bipolare Pfannen verwendet [9]. Generell liegt die Luxationsrate nach proximaler Femurresektion relativ hoch bei 37 % [21]. In einer weiteren Studie wurde zur Stabilisierung der Weichteile ein Trevira-Mesh eingesetzt, ein synthetisches Material mit hoher Reißfestigkeit und biokompatiblen Eigenschaften. Dieses ermöglicht eine zirkumferente Refixation der Muskulatur, insbesondere des M. iliopsoas und der pelvitrochantären Muskulatur [22]. Menendez et al. [23] zeigen, dass die Verwendung bipolarer Kopfsysteme das Luxationsrisiko um das Dreifache reduziert im Vergleich zur konventionellen Hüfttotalendoprothese.

### Weichteilrefixation und Prothesenstabilität

Farid et al. untersuchten verschiedene Techniken der Abduktorenrefixation bei 52 Patienten mit zementierten Prothesen. In 36 Fällen wurden die Abduktoren direkt mit nichtresorbierbaren Nähten an die Prothese angenäht. In 11 Fällen erfolgte zusätzlich eine Tenodese an das iliotibiale Band. Bei 5 Patienten wurde ein Trochanterrest mit Draht, Kabel oder einer Trochanterklammer refixiert. Alle drei Methoden zeigten eine gute funktionelle Wiederherstellung [24].

### Infektionsrisiko und Wundmanagement

Onkologische Patienten haben ein besonders hohes Risiko für infektiöse Komplikationen, das zwischen 1,2 und 19,5 % liegt, insbesondere nach einer präoperativen Strahlentherapie. Eine verzögerte Wundsekretion stellt mit einer Rate von 11–31 % einen wichtigen Risikofaktor für eine periprothetische Infektion (PJI) dar, wie Heinrichs, Guzik, Rowell und Jeys berichten.

Hettwer et al. verglichen zwei Verschlussmethoden und stellten fest, dass ein okklusiver Wundverschluss mit intradermalen Nähten, Steri-Strips™ und Hautkleber zu einer schnelleren Wundheilung (durchschnittlich 3,4 vs. 6,7 Tage) und einer kürzeren Hospitalisierung (6,3 vs. 8 Tage) führt [25]. Eine retrospektive Studie zeigte zudem, dass eine Drainagedauer von mehr als 7 Tagen mit einer verlängerten Antibiotikaprophylaxe (durchschnittlich 8,7 Tage) und einer längeren Krankenhausverweildauer (durchschnittlich 10,2 Tage) assoziiert war [20].

### Langfristige funktionelle Ergebnisse

Die durchschnittliche Überlebenszeit nach einer modularen Endoprothesenimplantation beträgt 2,5 Jahre [9]. Eine Langzeitstudie ergab, dass Patienten 13,2 Jahre nach dem Eingriff über eine effiziente Gehfähigkeit verfügten und am häuslichen sowie gesellschaftlichen Leben teilnehmen konnten. Ihre funktionellen Ergebnisse waren vergleichbar mit denen von Patienten nach einer konventionellen Hüfttotalendoprothese [26].

## Fazit für die Praxis

Präoperative Planung:

- Strenge Indikationsstellung (Lebenserwartung > 3 Monate, gute Allgemeinzustand).
- Umfassende Bildgebung zur Gefäß- und Weichteilplanung.

Intraoperative Technik:

- Knochenqualität prüfen → zementierte Verankerung bei schlechter Knochenstruktur.
- Biopolare Pfannen bevorzugen, wenn keine Azetabulum-Beteiligung vorliegt (reduziert Luxationsrisiko).
- Weichteilstabilisierung:
  a) Refixation der pelvitrochantären Muskulatur essenziell für Gelenkstabilität.
  b) Verwendung eines Trevira-Meshs zur zirkumferenten Muskelrefixation.

Postoperatives Management:

- Risiko für periprothetische Infektionen minimieren → okklusiver Wundverschluss.
- Frühzeitige Mobilisation zur Reduktion thromboembolischer Komplikationen.
- Engmaschige Nachkontrolle, um Rezidive und Spätkomplikationen frühzeitig zu erkennen.

Diese Punkte dienen als praxisnahe Handlungsrichtlinien, um die Ergebnisse nach proximaler Femurresektion mit endoprothetischer Versorgung zu optimieren.

## Supplementary Information

Video zur proximalen Femurresektion mit endoprothetischer Rekonstruktion bei malignen Knochentumoren.

## Abkürzungen

EPR: Endoprothetische Rekonstruktion
PJI: Periprothetische Gelenksinfektion
